# Dissecting multi-photon resonances at the large hadron collider

**DOI:** 10.1140/epjc/s10052-017-5162-5

**Published:** 2017-09-08

**Authors:** B. C. Allanach, D. Bhatia, Abhishek M. Iyer

**Affiliations:** 1 Department of Applied Mathematics and Theoretical Physics, Centre for Mathematical Sciences, University of Cambridge, Wilberforce Road, Cambridge, CB3 0WA UK; 2 Department of Theoretical Physics, Tata Institute of Fundamental Research, Homi Bhabha Road, Colaba, Mumbai, 400 005 India

## Abstract

We examine the phenomenology of the production, at the 13 TeV Large Hadron Collider (LHC), of a heavy resonance *X*, which decays via other new on-shell particles *n* into multi-(i.e. three or more) photon final states. In the limit that *n* has a much smaller mass than *X*, the multi-photon final state may dominantly appear as a two-photon final state because the $\gamma$s from the *n* decay are highly collinear and remain unresolved. We discuss how to discriminate this scenario from $X \rightarrow \gamma \gamma$: rather than discarding non-isolated photons, it is better to relax the isolation criteria and instead form photon jets substructure variables. The spins of *X* and *n* leave their imprint upon the distribution of pseudo-rapidity gap $\Delta \eta$ between the apparent two-photon states. Depending on the total integrated luminosity, this can be used in many cases to claim discrimination between the possible spin choices of *X* and *n*, although the case where *X* and *n* are both scalar particles cannot be discriminated from the direct $X \rightarrow \gamma \gamma$ decay in this manner. Information on the mass of *n* can be gained by considering the mass of each photon jet.

## Introduction

**Fig. 1:**
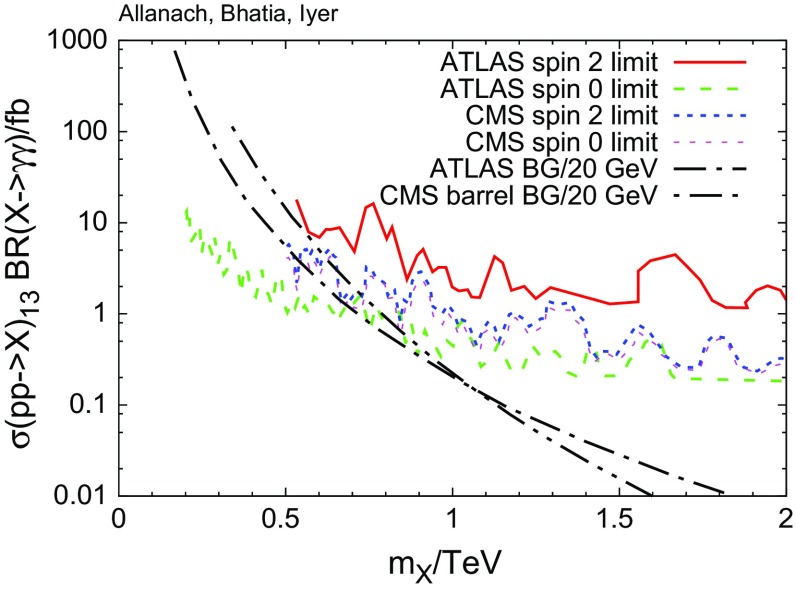
Upper limits on 13 TeV LHC di-photon resonance production and fitted backgrounds for the di-photon invariant mass spectrum. In the curves marked “limit”, we display the upper 95$\%$ confidence level limit on the cross section times branching ratio of a narrow resonance that decays into a two-photon final state. The ATLAS spin 0 limits were obtained from 15.4 fb$^{-1}$ of integrated luminosity [13], the ATLAS spin 2 limits came from 3.2 fb$^{-1}$ [14] under the assumption of a Randall Sundrum graviton [4], whereas the CMS limits come from a combination of 19.7 fb$^{-1}$ of 8 TeV collisions and 15.2 fb$^{-1}$ of 13 TeV collisions [3]. The *curves* labelled “BG” show central values of fitted di-photon mass spectra for 13 TeV LHC collisions in a 3.2 fb$^{-1}$ ATLAS analysis [14] and for a 12.9 fb$^{-1}$ CMS analysis  citeKhachatryan:2016yec where both photons end up in the barrel. The expected background (‘BG’) in each case is shown for a bin of width 20 GeV

The Standard Model (SM) of particle physics has been extensively tested to a great degree of accuracy. The discovery of a particle whose properties are so far consistent with those predicted for the SM Higgs boson have further fuelled the searches for Beyond the Standard Model (BSM) physics. The typical signatures employed in the search for these new physics scenarios involve different combinations of hard isolated photons, hard jets, hard isolated leptons and large missing transverse momentum. The presence of isolated leptons and isolated photons in a given final state is useful in significantly depleting SM backgrounds. The discovery of the Higgs boson in the di-photon channel [1, 2] has lead to an increased interest in the $\gamma \gamma$ final state. A hunt for a putative heavy resonance *X* enjoys enhanced sensitivity because SM backgrounds reduce quickly at larger di-photon invariant masses $m_{\gamma \gamma }$. Fits to the $m_{\gamma \gamma }$ distribution are obtained by both ATLAS and CMS by assuming simple functional forms. The central values of the fitted forms for 13 TeV LHC collisions are shown in Fig. 1. Such cross sections depend upon the cuts and details of the analysis in question, and we have plotted the central value of the cross section within bins of 20 GeV width obtained from the fit. The CMS analysis [3] displayed uncertainties, which are nonetheless small (even to the right-hand side of the curve they are small). Figure 1 also shows the 95$\%$ confidence level upper limits on the production cross section of a narrow resonance (we call this resonance *X*) that decays into a two-photon state from ATLAS and CMS. The resonant di-photon channel is then assumed to be

1 $$\begin{aligned} pp\rightarrow X+x\rightarrow \gamma \gamma +x, \end{aligned}$$

where *X* is electrically neutral and can either be a spin 0 or spin 2 resonance, whereas *x* is the remnant of the proton (for example, formed by spectator quarks), which tends to remain close to the beam-line and hence undetected. Below, we shall ignore *x*, since it is not relevant to the phenomenology that we discuss. There are quantitative differences if one takes the assumption of a broad resonance, but the picture is still roughly the same: for resonances of a mass larger than 1 TeV, the cross section times branching ratio upper limit from current experimental searches lies somewhere between 0.1 fb and 1 fb. It is clear from the figure that other assumptions as regards the resonance *X*, such as its spin, also affect the numerical value of the bound (this is because the acceptance of the signal changes). Assumptions as regards its production process, in particular, whether it is produced by quarks or gluons,^1^ also affect the signal acceptance and hence the bound.

Heavy scalars are can result from models which contain two higgs doublets [6], supersymmetric extensions of little Higgs models [7, 8] or extra-dimensional frameworks with bulk scalars [9]. Heavy gravitons can be attributed to the Kaluza Klein excitations of higher-dimensional gravity arising in either warped [4] or flat [10] geometries. The possibility of a spin 1 particle directly decaying to di-photons is forbidden by the Landau–Yang theorem [11, 12].

In some models, the heavy resonance *X* may decay into *nn* or $n \gamma$, where *n* is an additional light particle, may further decay into photons leading to a multi-photon^2^ final state. Examples of such models include hidden valley models [15, 16], the next-to-minimal supersymmetric standard model (NMSSM) [17] or Higgs portal scenarios [18]. There was an 8 TeV ATLAS search for a heavy resonance decaying into three and four photon states in Ref. [19]. For a mass of *n* greater than 10 GeV, and a scalar *X* of mass 600 GeV, the upper bound on cross section times branching ratios was 1 fb. For a $Z'$ particle of mass 100-1000 GeV, the bound on cross section times branching ratio into a three-photon final state (and *n* mass in the range 40–100 GeV) was found to be between 35 and 320 fb. However, in the limit where $m_n\ll m_X$, photons from *n* will be highly collimated, thereby creating the illusion of a di-photon final state from the detector point of view. Describing angles in terms of the pseudo-rapidity $\eta$ and the azimuthal angle around the beam $\phi$, the angular separation between two photons may be quantified by $\Delta R=\sqrt{(\Delta \eta )^2+(\Delta \phi )^2}$. Neglecting its mass, the opening angle between the two photons coming from a highly boosted on-shell *n* is

2 $$\begin{aligned} \Delta R=\frac{m_n}{\sqrt{z(1-z)}p_T(n)}, \end{aligned}$$

purely from kinematics (this was calculated already in the context of boosted Higgs to $b \bar{b}$ decays [20]), where *z* and $(1-z)$ are the momentum fractions of the photons.^3^ Thus,

3 $$\begin{aligned} \Delta R = \frac{m_n}{M_X}\frac{2 \cosh \eta (n)}{\sqrt{z(1-z)}}. \end{aligned}$$

In the limit $m_n/M_X\rightarrow 0$, $\Delta R\rightarrow 0$ and the two photons from *n* are collinear, appearing as one photon; thus several possible interpretations can be ascribed to an apparent di-photon signal.

Below, we shall examine the phenomenology of apparent $\gamma \gamma$ resonances, ignoring backgrounds. For this to be a good approximation, we require that the background is small compared to the signal cross section. Figure 1 shows that, for $m_X\mathop {{}_\sim }\limits ^{>}1200$ GeV, there is parameter space where this is the case, i.e. where $\sigma (pp \rightarrow X)\ BR(X \rightarrow \gamma \gamma )$ is well above the background but below the current experimental limits. The scenarios corresponding to different spins of *X* and *n* may be characterised by distributions of $\Delta \eta$ between the apparent di-photon states. Differences in the predicted $\Delta \eta$ distributions allows us to estimate the minimum number of events needed to discriminate between the different cases. In the event that the mass of the intermediate state *n* is not too small, such that the photons from it can often be resolved, the multi-photon topology can be distinguished from the di-photon topology using the substructure of photon jets [22, 23]. However, in the limit $m_n/m_X\rightarrow 0$, it is hard to resolve the photons from *n*.

There has been earlier work on heavy *X* spin discrimination in a truly di-photon final state: telling spin 0 from spin 2 [24–26]. However, our paper goes beyond these: we consider multi-photon cases which only appear to be di-photon cases at the first glance.

It will be useful for us to categorise models’ signatures into two classes: the first is multi-photon signals, where $m_n$ is large enough for the photons (from *n*) to be detected by different cells of the electromagnetic calorimeter, but small enough so that they produce the illusion of a single photon. The other category includes both the standard di-photon topology and the multi-photon topology in the limit $m_n/M_X\rightarrow 0$. Each apparent photon lies within a single cell of the electromagnetic calorimeter. These cases might be discriminated by photon-jet substructure properties. We shall use substructure variables to identify the fundamental nature of the topology and conventional kinematic variables to distinguish the different spin possibilities in each case.

The paper is organised as follows: in Sect. 2 we set up extensions to the SM Lagrangian which can predict heavy di-photon or multi-photon resonances. The finite photon resolution of the detector is discussed in Sect. 3. In Sect. 4, isolation criteria is removed and photon jets are adopted. Substructure and kinematic observables are then used to distinguish the different scenarios. In Sect. 5 we introduce the statistics which tell us how many measured signal events will be required to discriminate one set of spins from another, whereas we cover how one can constrain the mass of the intermediate particle *n* in Sect. 6. We conclude in Sect. 7. The appendix contains some details as regards model parameters.

## Model description

In this section we describe the minimal addition to the SM Lagrangian which can give rise to heavy resonant final states made of photons. We make no claims of generality: various couplings not relevant for our final state or production will be neglected. However, we shall insist on SM gauge invariance. Beginning with the di-photon final state, a minimal extension involves the introduction of a SM singlet heavy resonance *X*. We assume that any couplings of new particles such as the *X* (and the *n*, to be introduced later) to Higgs fields or $W^\pm , Z^0$ bosons are negligible. Equation (4) gives an effective field theoretic interaction Lagrangian for the coupling of *X* to a pair of photons, when *X* is a scalar (first line) or a graviton (second line). We have

4 $$\begin{aligned} \mathcal {L}_{X\,=\,\mathrm{spin~0}}^{int}= & {} - \eta _{GX}\frac{1}{4} G_{\mu \nu }^a G^{\mu \nu a} X - \eta _{\gamma X} \frac{1}{4} F_{\mu \nu } F^{\mu \nu } X, \nonumber \\ \mathcal {L}_{X\,=\,\mathrm{spin~2}}^{int}= & {} -\eta _{T\psi X}T_\mathrm{fermion}^{\alpha \beta } X_{\alpha \beta }\nonumber \\&-\eta _{TGX} T_\mathrm{gluon}^{\alpha \beta } X_{\alpha \beta } - \eta _{T\gamma X} T_\mathrm{photon}^{\alpha \beta } X_{\alpha \beta }, \end{aligned}$$

where $T^{\alpha \beta }_i$ is the stress-energy tensor for the field *i* and the $\eta _j$ are effective couplings of mass dimension -1. $F_{\mu \nu }$ is the field strength tensor of the photon (this may be obtained in a SM invariant way from a coupling involving the field strength tensor of the hypercharge gauge boson), whereas $G_{\mu \nu }^a$ is the field strength tensor of a gluon of adjoint colour index $a \in \{ 1,\ldots , 8 \}$. As noted earlier, the direct decay of a vector boson into two photons is forbidden by the Landau–Yang theorem [11, 12]. Since *X* is assumed to be a SM singlet, there are no couplings to SM fermions, which are in non-trivial chiral representations when it is a scalar.

The presence of an additional light scalar SM singlet in the theory (*n*), with masses such that $m_n<m_X$, opens up another decay mode: $X \rightarrow nn$. Lagrangian terms for these interactions are

5 $$\begin{aligned}&\mathcal {L}^{int}_{X\,=\,\mathrm{spin~0},n} = - \frac{1}{2}A_{Xnn} X n n , \nonumber \\&\mathcal {L}^{int}_{X\,=\,\mathrm{spin~2},n} = -\eta _{TnX} X_{\alpha \beta } T^{\alpha \beta }_n, \end{aligned}$$

where $A_{Xnn}$ has mass dimension 1. *n* may further decay into a pair of photons leading to a multi-photon final state through a Lagrangian term

6 $$\begin{aligned} \mathcal {L}^\mathrm{int}_{n\gamma \gamma }=-\frac{1}{4}\eta _{n\gamma \gamma } F_{\mu \nu } F^{\mu \nu } n. \end{aligned}$$

Although we assume that *n* is electrically neutral, it may decay to two photons through a loop-level process (as is the case for the Standard Model Higgs boson, for instance). Alternatively, if *X* is a spin 1 particle, it could be produced by quarks in the proton and then decay into $n \gamma$. The Lagrangian terms would be

7 $$\begin{aligned} \mathcal {L}^{int}_{X\,=\,\mathrm{spin~1},n}= & {} - \big (\lambda _{\bar{q} X q} \bar{q}_R \gamma _\mu X^\mu q_R \nonumber \\&+ \lambda _{\bar{Q} X Q} \bar{Q}_L \gamma _\mu X^\mu Q_L + H.c.\big )\nonumber \\&-\,\frac{1}{4}\eta _{nX\gamma } n{\tilde{X}}_{\mu \nu }F^{\mu \nu }, \end{aligned}$$

where $\lambda _i$ are dimensionless couplings, $q_R$ is a right-handed quark, $Q_L$ is a left-handed quark doublet and ${\tilde{X}}_{\mu \nu }=\partial _\mu X_\nu - \partial _\mu X_\nu$. The decay $X_{\mathrm{spin}=1}\rightarrow n \gamma$ would have to be a loop-level process, as explicitly exemplified in Ref. [21], since electromagnetic gauge invariance forbids it at tree level. A spin 1 particle may not decay into two identical spin 0 bosons due to Bose symmetry: the daughters must be symmetric under interchange, meaning they must have even orbital angular momentum *L*. Then it is impossible to conserve total angular momentum *J* since the initial state has $J=1$ and the final state has *J* even. Decays to non-identical spin 0 bosons are possible [27], but these are outside the scope of this paper.

For scalar *n*, then, we have a potential four photon final state if *X* is spin 0 or spin 2 and a potential three-photon final state if *X* is spin 1 as shown in Eq. (8):

8 $$\begin{aligned}&p~p\rightarrow X_{\mathrm{spin}=0,2}\rightarrow n n\rightarrow \gamma \gamma +\gamma \gamma \nonumber \\&p~p\rightarrow X_{\mathrm{spin}=1}\rightarrow n \gamma \rightarrow \gamma \gamma +\gamma . \end{aligned}$$

If the mass of the intermediate scalar *n* is such that $m_n \ll m_X$, its decay products are highly collimated because the *n* is highly boosted. It thereby results in a photon pair resembling a single photon final state. This opens up a range of possibilities with regards to the interpretation of the apparent di-photon channel. Above, we have assumed the intermediate particle *n* to be a scalar while considering different possibilities for the spin of *X*. Table 1 gives possible spin combinations for the heavy resonance *X* and the intermediate particle *n* leading to a final state made of photons. The third column gives the number of photons for each topology, grouped in terms of collimated photons that may experimentally resemble a single photon in the $m_n / m_X \rightarrow 0$ limit. The spin 1 *X* example was already proposed as a possible explanation [21] for a putative 750 GeV apparent di-photon excess measured by the LHC experiments (this subsequently turned out to be a statistical fluctuation).

**Table 1:** Different possibilities for spin assignments leading to an apparent di-photon state from other multi-photon final states. The one- or two-photon states have been grouped into terms which may only be resolved as one photon when $m_n/m_X$ is small

Spin of *X*	Spin of *n*	Number of photons
0	0	$\gamma \gamma$+$\gamma \gamma$
	2	$\gamma \gamma$+$\gamma \gamma$
1	0	$\gamma$+$\gamma \gamma$
	2	$\gamma$+$\gamma \gamma$
2	0	$\gamma \gamma$+$\gamma \gamma$
	2	$\gamma \gamma$+$\gamma \gamma$

In this work, we shall focus on the case where *n* is a scalar. However, the techniques developed in this paper can be extended to cases where *n* is spin 2 as well (but not spin 1, since $n \rightarrow \gamma \gamma$ would then be forbidden by the Landau–Yang theorem). In the next section we will describe the scenario under which the process in Eq. (8) can mimic a truly di-photon signal.

## The size of a photon

In a collider environment, any given process can be characterised by a given combination of final states. These final states correspond to different combinations of photons, leptons (electrons and muons), jets and missing energy. They can be distinguished by the energy deposited by them in different sections of the detector. In a typical high energy QCD jet, most of the final state particles (roughly 2/3) are charged pions whereas neutral pions make up much of the remaining 1/3 [22]. The constituents of a jet primarily deposit their energy in the hadronic calorimeter (HCAL) while the $\pi ^0\rightarrow 2\gamma$ decay of a neutral pion ensures that it shows up in the electromagnetic calorimeter (ECAL). Thus most of the constituents of the jet pass through the ECAL and deposit their energy in the HCAL. Photons and electrons deposit their energy in the ECAL, on the other hand. They can be distinguished by mapping the energy deposition to the tracker (which precedes the calorimeters). Apart from the tracker, electrons and photons are similar in appearance, from a detector point of view. Muons are detected by the muon spectrometer on the outside of the experiment.

We shall now go on to discuss the relevant parts of the detectors and experimental analyses. The actual construction and workings of the detector are of course much more detailed than we, outside of the experimental collaborations, have tools for dealing with. We therefore characterise the cuts and detector response in broad brush strokes. With this in mind, the experimental sensitivity to detect a single photon is subject to the following two criteria:

- *Dimensions of the ECAL cells*:  The ATLAS and CMS detectors have slightly different dimensions for the ECAL cells. ATLAS has a slightly coarser granularity with a crystal size of $(0.0256,\ 0.0254)$ in $(\eta ,\phi )$. In comparison, CMS has a granularity of $(0.0174,\ 0.0174)$ in $(\eta ,\ \phi )$. CMS and ATLAS have a layer in their electromagnetic calorimeters with finer $\eta$ segmentation (in ATLAS, this is called ‘layer 1’) but worse $\phi$ segmentation, which could also be employed in analyses looking for resonances into multi-photon final states. The level of ECAL modelling including this layer is beyond the scope of this paper, and so we do not discuss it further. However, we bear in mind that information from the layer 1 may be used in addition to the techniques developed in this paper. Any estimates of sensitivity (which come later) are therefore conservative in the sense that additional information from layer 1 could improve the sensitivity. High energy photons will tend to shower in the ECAL: this is taken into account by clustering the cells into cones of size $R_\mathrm{cone}=\Delta R=0.1$. Thus if two high energy signal photons are separated a distance $\Delta R<R_\mathrm{cone}$, they are typically not considered to be resolved by the ECAL since it could be a single photon that is simply showering.
- *Photon isolation*:  In ATLAS and in CMS, a photon is considered to be isolated if the magnitude of the vector sum of the transverse momenta ($p_T$) of all objects with $\Delta R \in [R_\mathrm{cone},\ 0.4]$ is less than 10$\%$ of its $p_T$. Qualitatively, this corresponds to the requirement that most of the energy is carried by the photon around which the cone is constructed. This criterion is required in order to distinguish a hard photon from a photon arising from a $\pi ^0$ decay.

**Fig. 2:**
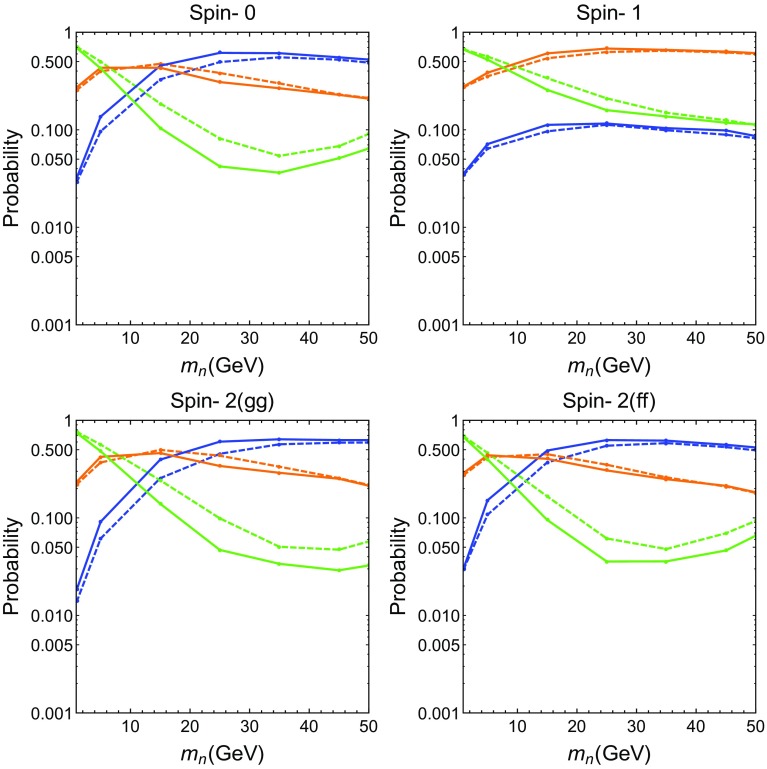
Probabilities of detecting different numbers of isolated, resolved photons for a 1200 GeV $X \rightarrow$ multi-photon decay as a function of $m_n$, the mass of the intermediate particle. We show the probabilities for zer (*blue*), one (*orange*) or two (*green*) photons for each *X* produced. The probabilities for detecting three or four isolated, resolved photons for the signal are very small for this range of $m_n$ and are not shown. *Solid lines* correspond to CMS, and *dashed lines* to ATLAS

However, it is possible that certain signal topologies may give rise final state photons that are separated by a distance $\Delta R \in [R_{cone},\ 0.4]$. For instance, consider the process given in Eq. (8). The particle *X* can either be a scalar or a graviton. For concreteness, let us assume that *n* is a scalar. In this case, a four photon final state resulting from $X \rightarrow nn \rightarrow \gamma \gamma + \gamma \gamma$ would appear to be a di-photon final state. However, as $m_n$ increases, eventually $\Delta R>0.4$ and the number of resolved final state photons will increase. Similar arguments hold for the case where particle *X* is a spin 1 state. For a given mass of *n*, the eventual number of detected, isolated and resolved photons depends on the granularity of the detector and is expected to be slightly different for both CMS and ATLAS.

To approximate the acceptance and efficiency of the detectors for our signal process, we perform a Monte Carlo simulation using the following steps:

- The matrix element for our signal process is generated in MadGraph5 aMC@NLO [28] by generating the Feynman rules for the process with FEYNRULES [29]. We set $\eta _i =\mathcal {O} (\text {20~TeV})^{-1}$ as specified in “Appendix”, $A_{Xnn}=M_X/100$ and $\lambda _i=0.5$ in the model file. MadGraph5 then calculates the width of the *X*: $\Gamma _X\sim 1-2$ GeV depending on the model, so the heavy resonance is narrow.^4^ Events are generated at 13 TeV centre of mass energy using the NNLO1 [30] parton distribution functions.
- For showering and hadronisation, we use PYTHIA 8.2.1 [31]. The set of final state particles is then passed through the DELPHES 3.3.2 detector simulator [32].

We use the DELPHES 3.3.2 isolation module for photons and we impose a minimum $p_T$ requirement of 100 GeV on each isolated photon.

Figure 2 shows the probabilities of detecting the different number of detected, resolved, isolated photons in the final state for a produced *X* for ATLAS (dashed) and CMS (solid). If $p_T(\gamma )<10$ GeV or $|\eta (\gamma )|>2.5$, DELPHES records a zero efficiency for the photon, and it is added to the ‘0 photon’ line. In the rest of the detector, DELPHES assigns between a 85$\%$ and a 95$\%$ weight for the photon (the difference from 100$\%$ is also added to the ‘0 photon’ line in the figure). A few of the simulated photons from the *X* additionally fail the $p_T>100$ GeV cut: these are not counted in the figure, and so the curves do not add exactly to 1.

The probabilities are shown for different possibilities of the spin of *X*, as shown by the header in each case. The bottom row corresponds to spin 2 when it is produced by *gg* fusion (left) and $\bar{q}q$ annihilation (right). Spin 1 corresponds to $X\rightarrow n \gamma \rightarrow \gamma \gamma + \gamma$, whereas the other cases all correspond to a $X \rightarrow nn \rightarrow \gamma \gamma + \gamma \gamma$ decay chain. The effective number of detected photons can be reduced by them not appearing in the fiducial volume of the detector (i.e. $|\eta (\gamma )|<2.5$), or by them not being isolated (in which case both photons are rejected) or resolved (in which they count as one photon). We note that, for each spin case, in the low $m_n$ limit, the *X* is most likely to be seen as two resolved, isolated photons because each photon pair is highly collimated.

**Fig. 3:**
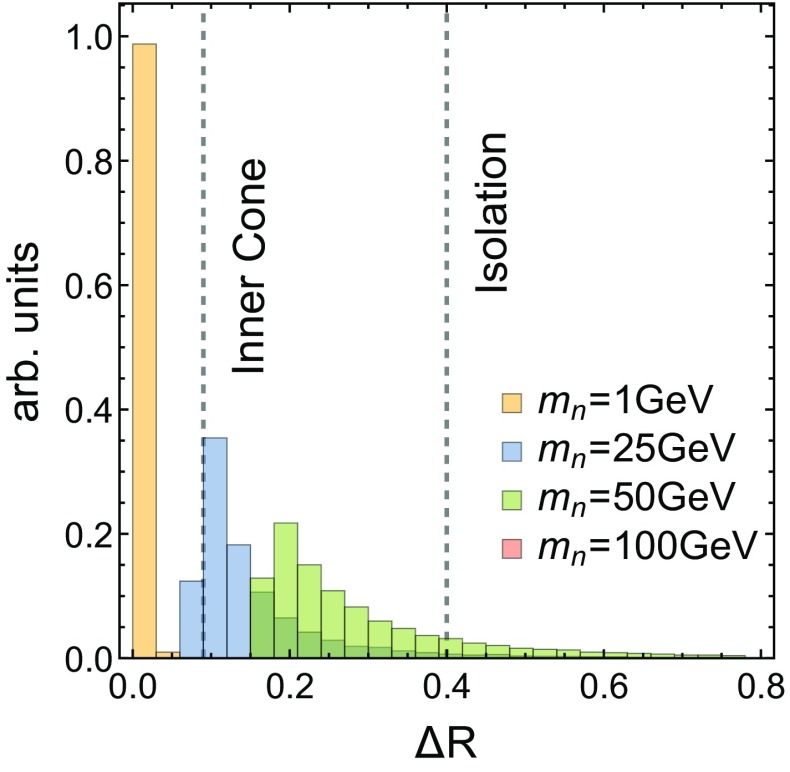
$\Delta R$ distribution for photon pairs originating from $n\rightarrow \gamma \gamma$ for different values of $m_n$. Photon pairs to the *left* hand side of the ‘ECAL Prescription’ *line* are considered to be one photon, whereas those between the ECAL prescription and the ‘Isolation’ *line* are rejected because of the photon isolation criteria

We note first that the probability for detecting zero, one or two resolved, isolated photons for the spin 2 case does not depend much on whether it is produced by a hard *gg* collision or a hard $$\bar{q} q$$ collision. An interesting trend is observed for the spin 0 and spin 2 cases, where the two-photon probability has a minimum at $$m_n\approx 40$$ GeV. At $$m_n=40$$ GeV, the photon pair from an *n* are often separated by $$\Delta R \in [R_\mathrm{cone},\ 0.4]$$ and fail the isolation criterion because the two photons have similar $$p_T$$. Figure 3 gives the distribution of $$\Delta R$$ between the photon pair coming from *n* as a function of its mass, and illustrates the preceding point. For light masses ($$m_n=1 $$ GeV) it is clear that both signal photons are within $$\Delta R<R_\mathrm{cone}$$. For intermediate masses $$m_n \in \{25,\ 50\}$$ GeV, most photons are within $$\Delta R \in [R_\mathrm{cone},\ 0.4]$$, whereas for $$m_n=100$$ GeV, a good fraction are already isolated photons, having $$\Delta R > 0.4$$. Using an estimate $$m_n \sim M_X \Delta R/4$$ from Eq. (3), we deduce that events with four isolated signal photons are expected to be evident only in the $$m_n \gtrsim 120$$ GeV region for $$M_X=1200$$ GeV.

The spin 1 case in comparison, has a significantly lower zero photon rate for $m_n < 50$ GeV, as the process is characterised by a single photon and two collimated photons. Thus, unless the single photon is lost in the barrel or lost because of tagging efficiency, it will be recorded even if the collimated photons fail the isolation criterion.

## Photon jets

Since we wish to describe collimated and non-isolated photons in more detail (since, as the previous section shows, these are the main mechanisms by which signal photons are lost), we follow Refs. [22, 23] and define photon jets. For this, we relax the isolation criteria and work with the detector objects, i.e. the calorimetric and track four vectors. The calorimetric four vectors for each event are required to satisfy the following acceptance criteria:

9 $$\begin{aligned} E_\mathrm{ECAL}>0.1 \text {~GeV},\quad E_\mathrm{HCAL}>0.5 \text {~GeV}, \end{aligned}$$

while only tracks with $p_T>2$ GeV are accepted. These calorimetric and track four vectors are clustered using FASTJET 3.1.3 [33] using the anti-$k_T$ [34] clustering algorithm with $R=0.4$. The tracks’ four vectors are scaled by a small number and are called ‘ghost tracks’: their directions are well defined, but this effectively scales down their energies to negligible levels to avoid over counting them (the energies are then defined from the calorimetric deposits). The photon-jet size $R=0.4$ is chosen to coincide with the isolation separation of the photon described in Sect. 3. The anti-$k_T$ clustering algorithm ensures that the jets are well-defined cones (similar to the isolation cone) and clustered around a hard momentum four vector, which lies at the centre of the cone. Thus for our signal events, the jets are constructed around the photon(s). These typically have a large $p_T$, since they are produced from a massive resonance.

Since these jets are constructed out of the calorimetric (and ghost track) four vectors, they constitute a starting point for our analysis. At this stage, while a QCD-jet (typically initiated by a quark or gluon) is on the same footing as a photon jet, they can be discriminated from each other^5^ by analysing different observables:

- *Invariant mass cut*:  We would demand the invariant mass of the two leading photon jets to be close to the mass of the observed resonance, reducing continuum backgrounds.
- *Tracks*:  QCD jets are composed of a large number of charged mesons which display tracks in the tracker before their energy is deposited in the calorimeter^6^ [35]. The track distribution for a QCD jet typically peaks at higher values of the number of tracks compared to a photon jet which peaks at zero tracks.
- *Logarithmic hadronic energy fraction* ($\log \theta _J$):  This variable is a measure of the hadronic energy fraction of the jet. For a photon jet most of the energy is carried by the hard photon(s). As a result, this jet will deposit almost all of its energy into the ECAL, which is in stark contrast with a QCD jet. This can be quantified by constructing the following substructure observable [22, 23]:
  10 $$\begin{aligned} \theta _J=\frac{1}{E^\mathrm{total}}\sum _iE_i^\mathrm{HCAL}, \end{aligned}$$
  where $E^\mathrm{total}$ is the total energy in the jet deposited in the HCAL plus that deposited in the ECAL, whereas $E_i^\mathrm{HCAL}$ is the energy of each jet sub-object *i* that is deposited in the HCAL. $\log (\theta _J)$ is large and negative for a photon jet, while it peaks close to $\log [2/3]=-0.2$ for a QCD jet, since charged pions constitute around (2 / 3) of the jet constituents. We would require the leading jet to have $\log (\theta _J)<-0.5$, corresponding to very low hadronic activity.

Under these cuts, the *QCD* fake rate should reduce to less than $10^{-5}$ [22, 23]. Removing photon isolation and instead describing the event in terms of photon jets is advantageous because it helps discriminate the standard di-photon decay in Eq. (1) from the decay to more than two photons in Eq. (8). However, it still fails in the limit $m_n/M_X \rightarrow 0$, as we shall see later. Taking photon jets as a starting point, we shall devise strategies where we may discern the nature of the topology and glean information as regards the spins of the particles involved.

**Table 2:** Cases to discriminate with a scalar *n* and a heavy resonance which is: scalar (*S*), spin 1 ($Z'$) or spin 2 (*G*). We have listed the main signal processes to discriminate between in the second column, ignoring any proton remnants. The notation used for a given model is *Xk*: $X=S,V,G$ labels the spin of the resonance and *k* denotes the number of signal photons at the parton level in the final state

Model	Process
*S*2	$pp \rightarrow S \rightarrow \gamma \gamma$
*S*4	$pp \rightarrow S \rightarrow nn \rightarrow \gamma \gamma +\gamma \gamma$
*V*3	$pp \rightarrow Z' \rightarrow n \gamma \rightarrow \gamma + \gamma \gamma$
$G2_{gg}$	$gg \rightarrow G \rightarrow \gamma \gamma$
$G2_{ff}$	$q \bar{q} \rightarrow G \rightarrow \gamma \gamma$
$G4_{gg}$	$gg \rightarrow G \rightarrow nn \rightarrow \gamma \gamma +\gamma \gamma$
$G4_{ff}$	$\bar{q} q \rightarrow G \rightarrow nn \rightarrow \gamma \gamma +\gamma \gamma$

### Nature of the topology

In this section we identify variables that aid in identifying the topology of the signal process and the spin of *X*. We begin by listing different cases we would like to discriminate between in Table 2. In the event of an observed excess in an apparent di-photon final state, we would relax the isolation criteria and define photon jets. Analysing the photon jets’ substructure will help measure the number of hard photons within each jet. The difference in substructure for a photon jet with a single hard photon as opposed to several hard photons can be quantified by [22, 23]

11 $$\begin{aligned} \lambda _J=\log \left( 1-\frac{p_{T_L}}{p_{T_J}}\right) . \end{aligned}$$

This can be understood as follows:

- Hard photon jets are re-clustered into sub-jets.
- $p_{T_L}$ denotes the $p_T$ of the leading sub-jet (i.e. the sub-jet with the largest $p_T$) within the jet in question, whilst $p_{T_J}$ is the $p_T$ of the parent jet.
- For a ‘single pronged’ photon jet, $p_{T_L}\sim p_{T_J}$. Thus $\lambda _J$ is negative, with a large magnitude.
- For a double-prong photon jet, $p_{T_L}<p_{T_J}$, resulting in $\lambda _J$ closer to zero than the single pronged jets. We expect a peak where $p_T(n)$ is shared equally between the two photons, i.e. $p_{T_L}/p_{T_J}=1/2$, or $\lambda _J=-0.3$.

There exist other substructure variables one could use in place of $\lambda _J$, such as *N*-Subjettiness [36, 37] or energy correlations [38] which are a measure of how pronged a jet is. Here, we prefer to use $\lambda _J$ because it is particularly easily implemented and understood, and it is robust in the presence of pile-up [39].

**Fig. 4:**
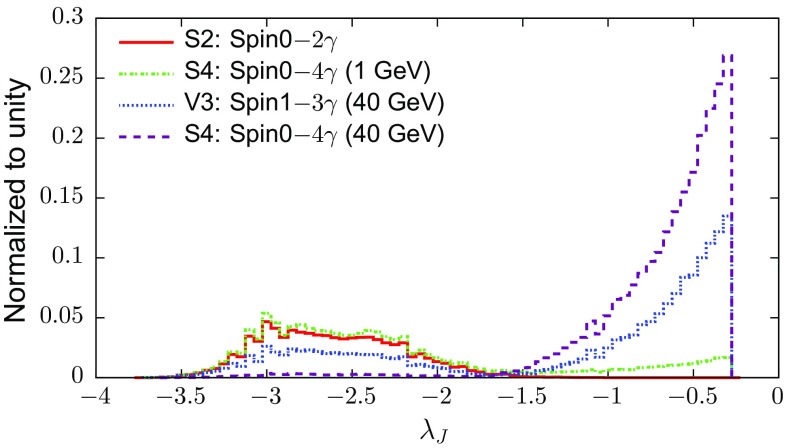
Distribution of $\lambda _J$ for *S*2 and some multi-photon topologies *S*4 for $m_n=1$ GeV and *V*3 and *S*4 for $m_n=40$ GeV in the ATLAS detector. Double photon jets dominantly appear at $\lambda _J \sim -0.3$. If a single hard photon in a jet radiates, it often appears in the bump $\lambda _J \in [-3.5,-2]$, but there is a possibility for the photon jet to really only contain one photon: here, $\lambda _J$ is strictly minus infinity. We do not show such events here on the figure, but they will count toward model discrimination

Figure 4 shows the distribution of $\lambda _J$ for the di-photon heavy resonance S2 (solid) and a multi^7^-photon S4 topology $m_n=1$ GeV (dot-dashed). It is evident from the figure that the $\lambda _J$ distribution is similar for the two cases, since they both peak at highly negative $\lambda _J$. This can be attributed to the fact that for such low masses of *n* in S4, the decay photons are highly collimated with $\Delta R < R_\mathrm{cell}$. They therefore should resemble a single photon. However, the appearance of a small bump like feature on the right of the plot for $m_n=1$ GeV S4 is interesting and unexpected prima facie since the opening angle between the photons in this case is less than the dimensions of an ECAL cell. However, this is explained by the fact that the energy of a photon becomes smeared around the cell where it deposits most of its energy. When a single (or two closely spaced photons) hit the centre of the cell, the smearing is almost identical for both cases. However, there exist a small fraction of cases for the collimated S4 topologies where the two photons hit a cell near its edge such that they get deposited in adjacent cells, leading to the small double-pronged jet peak at $\lambda _J=-0.3$. One would require both good statistics and a very good modelling of the ECAL in order to be able to claim discrimination of the two cases S2 and S4 (1 GeV), and for now we assume that they will not be. On the other hand, by the time that $m_n$ reaches 40 GeV, the multi-photon topologies V3 and S4 are easily discriminated from S2, due to the large double-photon peak at $\lambda _J=-0.3$. They should also be easily discriminated from each other since V3 has a characteristic double peak due to its $\gamma +\gamma \gamma$ topology.

**Fig. 5:**
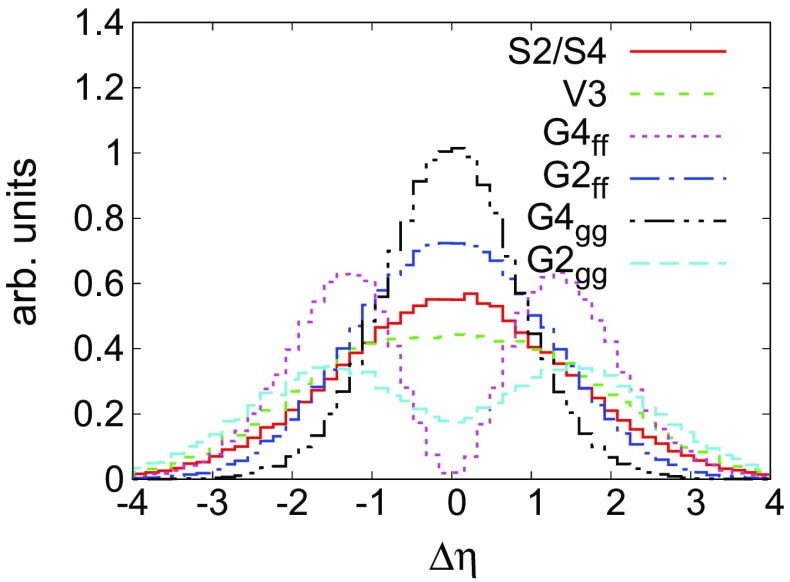
$\Delta \eta$ distribution between the two leading photon jets for the various models. There was very little difference between the S2 and S4 distributions by eye and so we have plotted them as one histogram

Using the $\lambda _J$ distribution of the apparent di-photon signal, we then segregate the different scenarios into two classes:

- *Case A: A peak in signal photons at* $\lambda _J=-0.3$  Here, the distribution in Fig. 4 points to the presence of intermediate particles *n* and intermediate masses (of say $m_n>15$ GeV) which lead to well-resolved photons inside the photon jet, e.g. V3 (40 GeV) and S4 (40 GeV) in Fig. 5. There are four possibilities under this category: $S4,V3,G4_{gg},G4_{ff}$ (see Table 2). Due to the double-peak structure *V*3 can be distinguished from $S4,G4_{ff},G4_{gg}$ using the $\lambda _J$ distribution.
- *Case B: No sizeable peak at* $\lambda =-0.3$  Here, we can either have S2 or intermediate particles *n* with a low mass. Most photon pairs coming from *n* appear as one photon since each from the pair hits the same ECAL cell. Thus, signal events resemble a conventional di-photon topology. All seven cases in Table 5 ($S2,S4,V3,G2_{gg},G4_{gg},G2_{ff},G4_{ff}$) can lie in this category, depending on $m_n/M_X$.

Once the nature of the topology is confirmed by the $\lambda _J$ distribution (i.e. a classification into case A or B), we then wish to determine the spin of the resonance *X* responsible for the excess.

**Fig. 6:**
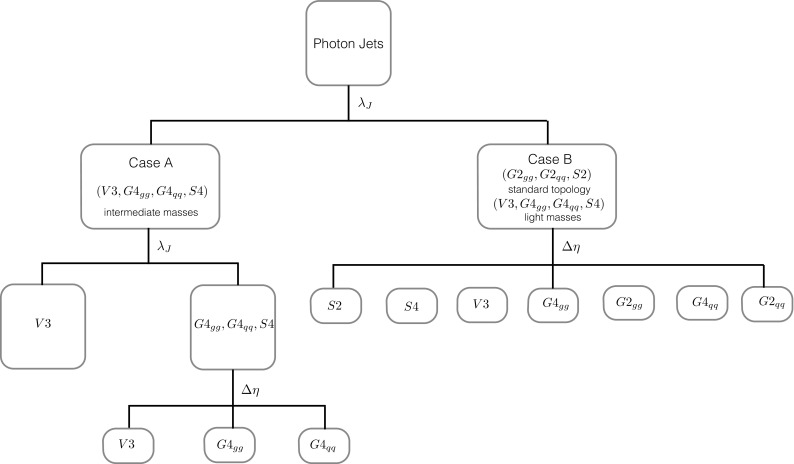
Flow chart representing the analysis strategy, beginning with photon jets, to discern the spin of the parent resonance *X*. After defining photon jets, the $\lambda _J$ distribution is used to select different possibilities: *Case A* where the $\lambda _J$ distribution indicates the presence of intermediate *n* particles in the decay with an intermediate mass. *Case B* indicates that either the intermediate particles are very light or absent. A double-bump structure in the $\lambda _J$ distribution indicates the spin 1 (*V*3) topology

Consider case A for instance: as shown in Fig. 6, the three remaining scenarios in case A, $S4,G4_{ff},G4_{gg}$, can be distinguished from one another by constructing the $\Delta \eta$ distribution between the leading signal photon jets. We classify $\Delta \eta$ for a given scenario as either central (peaking at zero) or non-central (two distinct peaks away from zero) as shown in Table 3. We show the various distributions in Fig. 5.

In the case where two scenarios can have the same $\Delta \eta$ distribution classification (e.g. *S*4 and $G4_{gg}$), one must examine differences in the precise shapes of these distributions to distinguish them. This will be discussed in the next section.

**Table 3:** Classification of the $\Delta \eta$ distributions of models (listed in Table 2) as either central or non-central

Model	*S*2	*S*4	*V*3	$G4_{gg}$	$G2_{ff}$	$G2_{gg}$	$G4_{ff}$
$\Delta \eta$	Central					Non central	

In case B, all seven models listed in Table 5 are possibly indicated if $m_n/M_X$ is very small. As shown in Fig. 6, $\Delta \eta$ will be needed to distinguish the various models.

## Spin discrimination

The discussion in the previous section illustrates the role of the substructure variables $\lambda _J$ and $\Delta \eta$. While $\lambda _J$ is useful in determining whether a given process results in well-resolved photons in the calorimeter, $\Delta \eta$ helps discriminate the different spin hypotheses from one another. The signal $\Delta \eta$ distribution changes depending upon which spins are involved in the chain and they are invariant with respect to longitudinal boosts. They should therefore be less subject to uncertainties in the parton distribution functions (PDFs), which determine the longitudinal boost in each case.^8^

We wish to calculate how much luminosity we expect to need in order to be able to discriminate the different spin possibilities in the decays, i.e. the different rows of Table 2. For this purpose, we assume that one particular hypothesis $H_T$, is true. Following Ref. [40] (which did a continuous spin discrimination analysis for invariant mass distributions of particle decay chains and large *N*), we require *N* signal events to disfavour a different spin hypothesis $H_S$ to some factor *R*. We solve

12 $$\begin{aligned} \frac{1}{R} = \frac{p (H_S| N \text {~events from~}H_T)}{p(H_T | N \text {~events from~}H_T)} \end{aligned}$$

for *N*, for some given *R* (here we will require $R=20$, i.e. that some spin hypothesis $H_S$ is disfavoured at 20:1 odds over another $H_T$). We are explicitly assuming that background contributions *B* are negligible to make our estimate, but in practice, they could be included in the $\Delta \eta$ distributions in which case $H_S \rightarrow H_S + B$ and $H_T \rightarrow H_T + B$ in Eq. (12).

We characterise the ‘*N* events from $H_T$’ by the values of a particular observable (or set of observables) $o_i$. In the present paper, we shall consider the pseudrapidity difference $\Delta \eta$ between the leading and next-to-leading photon jet, $o_i^{(T)}$ (for $i \in \{ 1,2,\ldots ,N\}$) that are observed in those events, although the observables could easily be extended to include other observables, for example $\lambda _J$. By Bayes’ theorem, we rewrite Eq. (12) as

13 $$\begin{aligned} \frac{1}{R}= & {} \frac{p(H_S)}{p(H_T)} \frac{p(N \text {~events~from~}H_T| H_S)}{p(N \text {~events from~}H_T | H_T)}\nonumber \\= & {} \frac{p(H_S)}{p(H_T)} \frac{\prod _{i=1}^N p(o^{(T)}_i | H_S)}{\prod _{i=1}^N p(o_i^{(T)} | H_T)}. \end{aligned}$$

Binned data measured in the *o* distribution $\{ n_j^{(T)} \}$ (for $j \in \{ 1,2,\ldots ,K\}$, *K* being the number of bins), will be Poisson distributed^9^ based on the expectation $\mu _j^{(X)}$ for bin *j*:

14 $$\begin{aligned} p(n_j | H_X) = \text {Pois}(n_j | \mu _j^{(X)}), \end{aligned}$$

where $X \in \{S, T\}$ and $\text {Pois}(n | \mu ) = \frac{\mu ^n e^{-\mu }}{n!}$. Substituting this into Eq. (13), we obtain

15 $$\begin{aligned} \log \left( \frac{1}{R}\right)= & {} \log \left( \frac{p(H_S)}{p({H_T})} \right) \nonumber \\&+ \sum _{j=1}^K \left[ n_j^{(T)} \log \frac{\mu _j^{(S)}}{\mu _j^{(T)}} + \mu _j^{(T)} - \mu _j^{(S)}\right] , \end{aligned}$$

where $\mu _j^{(T)}$ is the expectation of the number of events in bin *j* from $H_T$ and $n_j^{(T)}$ is a random sample of observed events obtained from $p(n_j | H_T)$. There is a (hopefully small) amount of information lost in going between unbinned data in Eq. (13) and binned data in Eq. (15). The first term on the right-hand side contains the ratio of prior probabilities of $H_T$ and $H_S$: this ratio we will set to one, having no particular a priori preference. Then taking the expectation over many draws, $\langle n^{(T)}_j \rangle = \mu ^{(T)}_j$ and so

16 $$\begin{aligned} \log \left( \frac{1}{R}\right) = \sum _{i=1}^K \left[ \mu ^{(T)}_j \log \frac{\mu _j^{(S)}}{\mu _j^{(T)}} + \mu _j^{(T)} - \mu _j^{(S)}\right] . \end{aligned}$$

We notice that Eq. (16) is not antisymmetric under $T \leftrightarrow S$, but this is expected since we assume that $H_T$ is the true hypothesis, in contrast to $H_S$. As the data come in, at some integrated luminosity, the distribution will be sufficiently different from the prediction of some other hypothesis, $H_S$, to discriminate against it at the level of 20 times as likely. Each term on the right-hand side is proportional to the collected integrated luminosity $\mathcal L$,

17 $$\begin{aligned} \mu _j^{(X)}=\mathcal L \sigma _\mathrm{tot}^{(X)} \epsilon ^{(X)}_j, \end{aligned}$$

where $\sigma _\mathrm{tot}^{(X)}$ is the assumed total signal cross section (i.e. the *X* production cross section) before cuts for $H_X$ and $\epsilon _j^{(X)}$ is the probability that a signal event makes it past all of the cuts and into bin *j*, under hypothesis *X*. Assuming that $\sigma _\mathrm{tot}^S=\sigma _\mathrm{tot}^T \equiv \sigma _\mathrm{tot}$, we may solve Eqs. 16 and 17 for ${ N}_R=\mathcal L \sigma _\mathrm{tot}$, the expected number of total signal events required to disfavour $H_S$ over $H_T$ to an odds factor of *R*:

18 $$\begin{aligned} N_R = \frac{\log R}{\sum _{j=1}^K \left[ \epsilon ^{(T)}_j \log \frac{\epsilon _j^{(T)}}{\epsilon _j^{(S)}} + \epsilon _j^{(S)} - \epsilon _j^{(T)}\right] }. \end{aligned}$$

One property of this equation is that if $\epsilon ^{(T)}_j=\epsilon ^{(S)}_j$ $\forall$ *j*, then ${\mathcal L}_R \rightarrow \infty$. This makes sense: there is no luminosity large enough such that it can discriminate between identical distributions. Equation (18) works for multi-dimensional cases of several observables: one simply gets more bins for the multi-dimensional case. If one works in the large statistics limit, for continuous data (rather than binned data), one obtains a required number of events that is related [40] to the Kullback–Leibler divergence instead [41]. The Kullback–Leibler divergence is commonly used when one has analytic expressions for distributions of the observables (see Ref. [40]), and it has the advantage of utilising the full information in *o*. We do not have analytic expressions, partly because they depend upon parton distribution functions, which are numerically calculated. Our method loses some information by binning, but it has the considerable advantage that it includes kinematical selection and detector effects (all contained within the $\epsilon _j$). Equation (18) has the property that if one halves the total *X* production cross section, one requires double the luminosity to keep the discrimination power (measured by *R*) constant.

Since we shall estimate $\epsilon ^{(X)}_j$ numerically via Monte Carlo event generation, there is a potential problem we have to deal with: a bin might end up with no generated events and so one encounters divergences from the logarithm in the denominator of Eq. (18). This is due, however, to not using enough Monte Carlo statistics, where *M* signal events are simulated in total for each parameter choice and for each hypothesis pairing. We restrict the range of *o* and use large enough Monte Carlo statistics ($M=200000$) such that no bins (which are set to be wide enough) contain zero events.

### Event selection and results

Using the statistic developed in Eq. (18), we first discriminate Case A from B defined in Sect. 4.1. Thus, in the event of an apparent di-photon excess in a certain invariant mass bin say $m^{(0)}_{\gamma \gamma }$, we propose the following steps:

- We relax the isolation criteria and re-analyse the events by constructing photon jets.
- The invariant mass $m_{j_1 j_2}$ of the two leading photon jets for each events are required to lie around $m^{(0)}_{\gamma \gamma }$: we require $1100<m_{j_1 j_2}/\text {GeV}<1300$.
- Photon jets from pions are eliminated by requiring that leading jets have no tracks ($n_T=0$) and by requiring $\log \theta _J<-0.5$. We also take into account the photon conversion factor. This depends on whether the photon converts before or after exiting the pixel detector. This conversion probability is a function of the number of radiation lengths (*a*) a photon passes through before it escapes the first pixel detector and is given by [22]
  19 $$\begin{aligned} P(\eta )=1-\exp \left( -\frac{7}{9}a(\eta )\right) . \end{aligned}$$
  We approximate this by an $\eta$ independent conversion probability $P(\eta )=0.2$.
- The substructure of each jet is analysed using $\lambda _J$ to determine whether it is in Case A or B.

Figure 6 gives a pictorial representation of these steps. We use $m_n=40$ GeV and $m_n=1$ GeV as examples for the model hypotheses to be tested. We simulate $2\times 10^5$ events for the topologies predicted by $H_T$ and $H_S$ and compute $\lambda _J$ for all events which pass the basic selection criteria. To avoid any zero event bins, $\lambda _J$ is binned between $[-4,0]$ with a bin size of 0.6 and the efficiency for each particular bin is extracted for both distributions from the simulation. Owing to the distinct nature of the $\lambda _J$ distribution for the two cases, 3–4 events is sufficient to discriminate between case A and case B. The $m_n=1,40$ GeV cases both have a post-cut acceptance efficiency of ${\sim }55\%$. For a cross section of 0.5 fb, we can accumulate some five signal events with $\sim$18 fb$^{-1}$ of integrated luminosity. Once the nature of the topology (corresponding to a given case) is identified, our next step is to discriminate the different possibilities within it. The two scenarios are handled independently as follows:

**Case A**  In this case there are only four possibilities corresponding to a multi-photon topology (i.e. proceeding through an intermediate *n*). As discussed earlier, we do not impose the requirement of two isolated photons, since the photons from *n* tend to fail isolation cuts. We compute $\Delta \eta$ between the two leading photon jets. In order to discriminate V3 from the other cases, the twin-peaked structure of *V*3 under $\lambda _J$ (as shown in Fig. 4) can be employed to discriminate it collectively from $S4,G4_{gg},G4_{ff}$. In this case one requires a minimum of 20 signal events to disfavour the other three at a 20 : 1 odds. All samples are characterised by a minimum of ${\sim }55\%$ acceptance efficiency. With this information, one can disfavour $S4,G4_{gg},G4_{ff}$ in favour of *V*3 with $\sim$ 72 fb$^{-1}$ of integrated luminosity for a 0.5 fb signal cross section.

$S4,G4_{gg},G4_{ff}$ can then be discriminated from one another using $\Delta \eta$ between the two leading jets. Table 4 computes the minimum number events required for pairwise discrimination of the three cases for $m_n=40$ GeV and is computed using Eq. (18) To avoid zero event bins in the $\Delta \eta$ distribution, we restrict the a priori range of $|\Delta \eta | \in [-5,5]$ to $[-4,4]$. As shown in Table 4, disfavouring *S*4 as compared to $G4_{gg}$ constitutes the largest expected number of required signal events *i.e.* 29. This can be achieved with a luminosity of $\sim$ 105 fb$^{-1}$. Thus in the event of a discovery corresponding to Case A, it is possible to get exact nature of the spin of *X* within 105 fb$^{-1}$ of data.

**Table 4:** Spin discrimination: $N_R = {\mathcal L} \sigma _\mathrm{tot}^{(X)}$, the expected number of total signal events required to be produced to discriminate against the ‘true’ row model versus a column model by a factor of 20 at the 13 TeV LHC for $m_n=40$ GeV

$N_R$	*S*4	$G4_{gg}$	$G4_{ff}$
*S*4	$\infty$	22	13
$G4_{gg}$	29	$\infty$	4
$G4_{ff}$	19	5	$\infty$

**Case B**  This constitutes the more complicated of the two cases. Since the two hard photons inside the photon jet for the multi-photon topologies cannot be well resolved, the substructure is similar to the conventional single photon jet from the standard di-photon topology. Thus there are more cases to distinguish in this case. We compute the $\Delta \eta$ between the leading two jets of the event. To avoid zero event bins in the $\Delta \eta$ distribution, we restrict the a priori range of $\Delta \eta$ from $[-5,5]$ to $[-4,4]$.

The signal models here are characterised by an acceptance efficiency of at least $55\%$. Using the cross section of 0.5 fb, we find that the cases *S*2 and *S*4 are virtually indistinguishable owing to the similar shapes of their $\Delta \eta$ distributions. They thus cannot be distinguished on the basis of the $\Delta \eta$ distribution. However, as shown in Fig. 4, the presence of a secondary bump for the collimated case will help in distinguishing these two cases. In this case, the same technology we have developed for the $\Delta \eta$ distribution could be employed for the $\lambda _J$ distribution.

Distinguishing *S*2, *S*4 from *V*3 requires a maximum expected number of events of 250–300. This is achievable with 1.1 ab$^{-1}$ of integrated luminosity, assuming an acceptance of ${\sim }55\%$ and a signal production cross section of 0.5 fb. Distinguishing scenarios like *S*2 from $G4_{ff}$ or $G4_{gg}$ requires 23 events or less: these could be discriminated with $\sim$84 fb$^{-1}$ for our reference cross section of 0.5 fb, whereas the rest of the pairs of spin hypotheses can be distinguished within 364 fb$^{-1}$ of data (Table 5).

**Table 5:** Spin discrimination of two models: $N_R = {\mathcal L} \sigma _\mathrm{tot}^{(X)}$, the expected number of total signal events required to be produced to discriminate against the ‘true’ row model versus a column model by a factor of 20 at the 13 TeV LHC for $m_n=1$ GeV

$N_R$	*S*2	*S*4	*V*3	$G2_{gg}$	$G4_{gg}$	$G2_{ff}$	$G4_{ff}$
*S*2	$\infty$	${>}2000$	272	27	15	91	14
*S*4	${>}2000$	$\infty$	255	26	15	96	13
*V*3	260	248	$\infty$	54	9	37	21
$G2_{gg}$	32	31	65	$\infty$	5	13	38
$G4_{gg}$	23	24	14	6	$\infty$	54	4
$G2_{ff}$	102	110	44	12	40	$\infty$	8
$G4_{ff}$	19	18	28	37	5	12	$\infty$

## Mass of the intermediate scalar

**Fig. 7:**
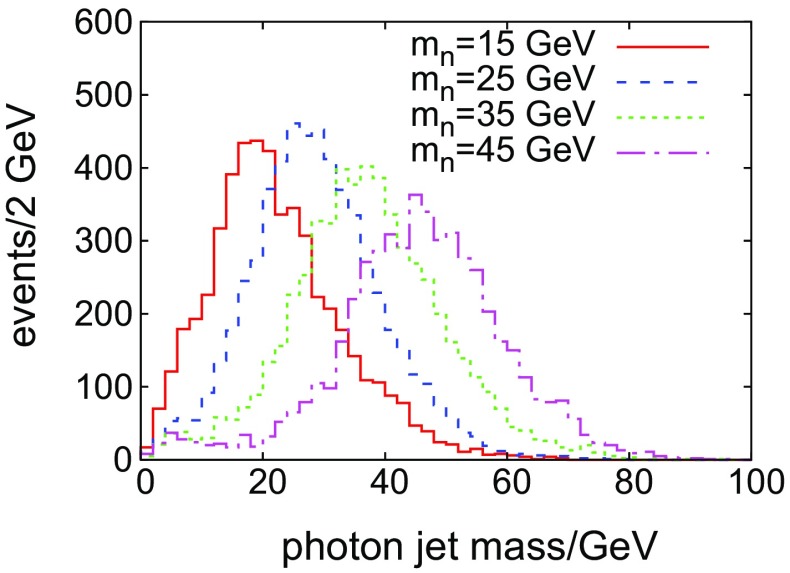
Comparison of the S4 photon-jet mass distributions for the leading photon jets and various $m_n$

A multi-photon topology is indicative of the presence of two scales in the theory: $m_X$ and $m_n$. While the scale of the heavier resonance is evident from the apparent di-photon invariant mass distribution, extracting the mass of the lighter state may be more difficult. From Fig. 2, we see that, for low to intermediate masses, one does not obtain isolated photons from *n* which may be used to reconstruct its mass. We therefore examine the invariant mass of photon jets. The decay constituents of *n* retain its properties such as its $p_T$, pseudo-rapidity $\eta$, mass *etc.* Figure 7 shows a comparison of the mass of the leading jet for *S*4 and a few different values of $m_n$.

The peak of each distribution, which can be fitted, clearly tracks with the mass of *n*. Using an estimate based on the statistical measure introduced in Sect. 5, we calculate that 25 signal events would be required to discriminate the 35 GeV from the 45 GeV hypothesis, for instance: i.e. $\sim$91 fb$^{-1}$ of integrated luminosity and a signal cross section of 0.5 fb. Thus, for intermediate masses and reasonable amounts of integrated luminosity, a fit to the peak should usefully constrain $m_n$, at least for $m_n\gtrsim 10$ GeV.

## Conclusion

In the event of the discovery of a resonance at high di-photon invariant masses, it will of course be important to dissect it and discover as much information as regards its anatomy as possible. Here, we have provided a case for Refs. [22, 23], where photon jets, photon sub-jets and simple kinematic variables were defined that might provide this information. The apparent di-photon signals may in fact be multi-photon (i.ė greater than two photons), where several photons are collinear, as is expected when intermediate particles have a mass much less than the mass of the original resonance. We identified useful variables for this purpose: the pseudo-rapidity difference between the photon jets helps discriminate different spin combinations of the two new particles in the decays. We quantify an estimate for how many signal events are expected to be required to provide discrimination between different spin hypotheses, setting up a discrete version of the Kullback–Leibler divergence for the purpose. For the discovery of a 1200 GeV resonance with a signal cross section of 0.5 fb, many of the spin possibilities can be discriminated within the expected total integrated luminosity expected to be obtained from the LHC. A simple sub-jet variable $\lambda _J$ provides a good discriminant between the di-photon and multi-photon cases. The invariant mass of the individual photon jets provides useful information as regards the intermediate resonance mass.

We hope that our study motivates work from the experimental collaborations, which have access to detailed detector information. For example, it would be interesting to see how much ‘layer 1’ of ATLAS’ ECAL would help verify the very light *n* cases. Also, photon conversion rates would be different for two almost collinear photons and for a single photon, providing another possible tool for diagnosing multi-photon final states.

## Appendix: Signal model parameters

We now detail the model parameters picked for each case for our numerical simulations. Firstly we specify the $X\rightarrow \gamma \gamma$ case, where we choose $\eta ^{-1}_{GX}=40$ TeV and $\eta ^{-1}_{\gamma X}=80$ TeV. When *X* is spin 2 and we consider fermion anti-fermion production, $\eta ^{-1}_{T\psi X}=\eta ^{-1}_{T \gamma X}=40$ TeV. When *X* is spin 2 and we consider gluon gluon production, $\eta ^{-1}_{T G X}=\eta ^{-1}_{T \gamma X}=80$ TeV.

When instead we consider intermediate scalar *n* particles in the decays of *X*, we fix $\eta _{n \gamma \gamma }^{-1}=\eta _{GX}^{-1}=20$ TeV for the spin 0 *X* case. For spin 1 *X*, $\eta _{n X \gamma }=0.3/(10 \text {~TeV})$ and $\eta _{n \gamma \gamma }^{-1}=10$ TeV. For spin 2 *X* and fermion anti-fermion production, $\eta ^{-1}_{Tn X}=\eta _{n \gamma \gamma }^{-1}=10$ TeV and $\eta ^{-1}_{T \psi X}=20$ TeV. For spin 2 *X* and glue glue production, $\eta ^{-1}_{Tn X}=\eta _{n \gamma \gamma }^{-1}=20$ TeV and $\eta ^{-1}_{TG X}=40$ TeV.

## Footnotes

1 For example, the spin 2 Randall–Sundrum graviton [4] has a well-defined ratio of production cross sections between *gg* and $q \bar{q}$, depending upon its mass [5].

2 In the present paper, whenever we refer to multi-photon final states, we refer to three or more photons.

3 The decay is strongly peaked towards the minimum opening angle $\Delta R=2 m_n/p_T$ [21].

4 The light resonance is also narrow, since $\Gamma _n = m_n^3 |\eta _{n \gamma \gamma }|^2 / (64 \pi )$.

5 Here we have not implemented such cuts, since we only simulated signal.

6 A gluon initiated jet typically has a larger track multiplicity than a quark initiated jet.

7 In this article, we refer to three or more hard signal photons as a multi-photon state.

8 We note that whether the photon is in the fiducial volume or not does depend upon the longitudinal boost, and is therefore subject to PDF errors.

9 As argued above, we work in kinematic régimes where backgrounds can be neglected. We are also neglecting theoretical errors in our signal predictions. It would be straightforward to extend our analysis to the case where some smearing due to theoretical uncertainties is included, where we would convolute Eq. (14) with a Gaussian distribution.
